# Radiation therapy trends following breast-conserving surgery in early-stage estrogen receptor-positive breast cancer: particularly in older patients more frequently omitted

**DOI:** 10.1016/j.breast.2026.104893

**Published:** 2026-08-05

**Authors:** Lotte Bouwman, Erik J. Blok, Astrid N. Scholten, Sabine Siesling, Johanneke E.A. Portielje, Gerrit-Jan Liefers

**Affiliations:** a Department of Surgery, Leiden University Medical Center, Leiden, the Netherlands; b Department of Medical Oncology, Leiden University Medical Center, Leiden, the Netherlands; c Department of Radiation Oncology, The Netherlands Cancer Institute, Amsterdam, the Netherlands; d Department of Research and Development, Netherlands Comprehensive Cancer Organisation (IKNL), Utrecht, the Netherlands; e Department of Health Technology and Services Research, Technical Medical Centre, University of Twente, Enschede, the Netherlands

**Keywords:** Breast cancer, Low-risk, Radiation therapy, de-escalation, Breast-conserving surgery

## Abstract

**Objectives:**

To evaluate nationwide trends in administration of (partial) breast radiation therapy (RT) following breast-conserving surgery (BCS) in women with early-stage, ER + breast cancer (BC).

**Methods:**

Women aged ≥50 years with ER+, HER2-, non-metastatic BC, treated between 2014 and 2024 with BCS were included. Eligible tumours were ≤1 cm grade 1 or 2, or ≤2 cm grade 1. Descriptive statistics and multivariable logistic regression were performed.

**Results:**

In total 21,418 patients were included. Endocrine therapy was administered in 3.5%.

Age related differences in RT administration were observed. In younger patients (50-69 years), RT omission increased from 1.8% (2014-2016) to 8.7% (2023-2024, p < 0.001). Among older patients (≥70 years), omission increased from 4.6% (2014-2016) to 55.3% (2020-2022) and 33.8% (2023-2024, p < 0.001).

RT type was comparable across age groups. Whole breast RT (WBRT) without boost was most common (>65%), followed by partial breast irradiation (PBI) (>21%). Over time, application of WBRT without boost decreased from 81.2% (2014-2016) to 46.5% (2023-2024, p < 0.001), while PBI increased from 4.3% (2014-2016) to 51.9% (2023-2024, p < 0.001). Age (OR 10.1) and year of diagnosis were strongly associated with RT omission.

**Conclusion:**

Younger patients received RT more often than older patients. Over time, WBRT remained the most common RT type, although its use decreased over time, while PBI increased. Omission of RT became more frequent, most markedly in older patients, partially driven by participation in a national trial on RT omission (TOP-1 study). Ultimately, RT was omitted in about one third of older patients, indicating an ongoing shift towards treatment de-escalation, individualized medicine or premature trial implementation.

## Highlights

• Younger patients were more often treated with radiation therapy (RT) after BCS.
• Whole-breast irradiation decreased over time, while partial-breast increased.
• Over the years omission of (partial) breast RT was more frequent in older patients.
• This trend may be explained by tailored care or premature study implementation.
• In subsequent years, omission of (partial) breast RT exceeded pre-study rates.

## 1 Introduction

The prevalence of invasive breast cancer (BC) has been rising over the past decades, with an incidence of around 15,600 women in the Netherlands in 2024 [1]. The majority of patients are aged 50 years and older and detection of early-stage BC occurs frequently between 50 and 75 years within the national screening program [[2], [3], [4]]. Prognosis and overall therapy benefit vary individually, depending on factors such as age, comorbidities, tumour type and stage, emphasizing the importance of individualization of treatment decisions [3,5].

One treatment option that allows for personalized treatment is (partial) breast radiation therapy (RT), which is standard of care after breast-conserving surgery (BCS) [6]. The primary goal of RT is to target microscopic residual (tumour) cells and, if applicable, ductal carcinoma in situ (DCIS) that may remain after surgery, enhancing local control and thereby reducing the risk of a local recurrence (LR) with a factor three to four [7]. The PRIME-II study has shown that postoperative whole-breast radiation therapy (WBRT), combined with endocrine therapy (ET), decreased the 5-year LR rate from 4.1% (without WBRT) to 1.3% [8]. The absolute benefit increased with longer follow-up. At 10 years, LR rate was 9.5% without WBRT versus 0.9% with WBRT [9]. These involved women aged ≥ 65 years with early-stage, hormone receptor-positive, axillary node negative BC, tumour diameter up to 3 cm and BCS with clear surgical margins. Similarly, previous studies have demonstrated improved LR-free survival when BCS is followed by RT [10,11].

RT also has negative effects, for example treatment burden and toxicity in organs at risk with WBRT [12,13]. WBRT with boost is associated with worse cosmetic outcomes and its use is, nowadays, mainly reserved for patients at high risk of recurrence [6,[14], [15], [16]]. Partial-breast irradiation (PBI) is indicated for patients at low risk of recurrence. PBI targets a more limited tissue volume than WBRT and trials have demonstrated less (acute) toxicity and non-inferiority, compared with WBRT [12,13,17,18].

From 2008 to 2016, the administration of RT after BC surgery increased in the Netherlands, partly driven by an increase in BCS [15]. However, after 2016, a trend of RT de-escalation has been observed in invasive BC [15]. This may be explained by better understanding of tumour biology, the lack of overall survival (OS) benefit from RT in clinically low-risk BC patients, and improved shared decision-making (SDM) [9,10,[19], [20], [21]]. Furthermore, Evers et al. stated that the decline in RT after 2016 can partly be attributed to the TOP-1 study (results pending, CCMO NL58117.058.16), a nationwide study that prospectively investigates the omission of postoperative RT without ET after BCS in women aged ≥ 70 years with estrogen receptor positive (ER+), non-metastatic BC, treated between 2017 and 2022. The observed decrease, however, exceeds the number of participants of the trial, suggesting a possible shift in nationwide clinical [6,15]. Notably, this RT decrease was observed in a population not routinely treated with ET according to the Dutch Breast Cancer Guidelines [6].

It is unclear whether this downward trend in RT has continued over time and if de-escalation was only observed in older patients or also in younger women. Therefore, this descriptive study primarily aims to gain insight into the administration of RT after BCS between 2014 and 2024, comparing younger (50–69 years) and older women (≥70 years) with low-risk BC. Secondly, different types (target volumes) of postoperative RT are compared. Finally, TOP-1 study inclusions, and the associated number of patients in whom RT was omitted, will be considered within the broader context of RT omission, thereby providing an up-to-date overview of postoperative RT trends in early-stage BC over the past decade in the Netherlands.

## 2 Methods

### 2.1 Study design and patient selection

This is a national, observational, cross-sectional study. Eligible women were aged 50 years or older at time of diagnosis, with histologically confirmed non-metastatic invasive BC, treated between 2014 and 2024. Furthermore, the tumour had to be ER+ and HER2-negative with a diameter of at maximum 1 cm (T1a or T1b) grade 1 or 2, or up to 2 cm (T1c), grade 1. Hormone receptors were considered positive if ≥ 10% of the tumour cells showed positive staining [6]. In addition, only BCS was allowed as surgical procedure and margins of the invasive component had to be free. Multiple breast-conserving procedures were allowed to achieve negative margins. The sentinel node (SN) had to be negative (N0 or N0itc). If DCIS was present alongside the invasive tumour, only clear surgical margins were permitted. Patients were excluded if they had BC in their medical history or in case of a bilateral tumour. Classification of tumours was done according to the TNM classification version 7 (2010-2016) or 8 (2017-2024) depending on year of diagnosis. The Bloom-Richardson scale was used for histological grading of the tumours.

The dataset consisted of clinical data with patient- and tumour characteristics and treatment regimen(s). These data were obtained from the Netherlands Cancer Registry (NCR) by data managers of the Netherlands Comprehensive Cancer Organisation (IKNL), who collected the data from individual patient records.

Patients were stratified by age in a younger (50-69 years old) or older (≥70 years) group, as well as by 10-year intervals for the analysis. To observe possible changes and trends in RT types and administration over time, patients were grouped by year of diagnosis (2014-2016, 2017-2019, 2020-2022 and 2023-2024). At last, a subgroup analysis was performed in patients aged ≥ 70 years to compare nationwide data with TOP-1 study inclusions (internal dataset, results pending, CCMO NL58117.058.16). This prospective single-arm study involved 46 hospitals in the Netherlands and included 1225 patients aged ≥70 years between 1 January 2017 and 31 December 2022. RT was omitted in all TOP-1 study participants, all of whom were also included in the obtained NCR dataset. The proportion of TOP-1 participants were presented as part of the total NCR study population.

### 2.2 Statistical analysis

Continuous variables were expressed as mean (standard deviation (SD)) or median (interquartile range (IQR)). Categorical variables were presented as absolute numbers and percentages. To compare patient characteristics and oncological parameters, descriptive statistics are used. The Chi-square test was used for categorical data and in case of small groups, the Fisher's exact test. For comparison of continuous variables, the Student's t-test or Mann-Whitney *U* test for independent groups was performed. Factors associated with omission of RT were identified using multivariable logistic regression analysis. Odds ratios (ORs), 95% confidence intervals (CIs) and p-values were calculated. A p-value of < 0.05 was considered statistically significant. Analyses were performed in R version 4.4.1 for Windows.

### 2.3 Ethical considerations

This study was performed as a non-WMO study. All is performed in accordance with ethical principles having their origin in the Declaration of Helsinki (64th version, October 2013). It is consistent with the International Conference on Harmonization (ICH)/Good Clinical Practice (GCP) E6(R2) guidelines and applicable regulatory requirements. The study was approved by the Privacy Review Board of the Netherlands Comprehensive Cancer Organisation (IKNL).

## 3 Results

From January 2014 to December 2024, 21,418 patients with early-stage BC met the inclusion criteria. Of all patients, 68.3% (n = 14,631) were aged 50-69 years and 31.7% (n = 6787) were aged 70 years or older (Table 1). All tumours were ER+ and ≤ 2 cm (pT1) as defined by the inclusion criteria. Progesterone receptor (PR) was positive in 83.8% of the tumours and 69.2% of tumours were grade one. These distributions were comparable between younger and older patients. Morphologically, invasive carcinoma of no special type was most prevalent in 81.2% of younger and 77.2% of older patients, followed by lobular carcinoma (7.7% and 8.4%, respectively). Mucinous carcinoma was relatively more prevalent among older patients (5.0%) compared with younger patients (2.0%).

**Table 1:** Baseline characteristics of younger women (50-69 years old) compared to older (≥70 years old) women.

Characteristic	Age 50-69 yrs N = 14,631^a^	Age ≥70 yrs N = 6,787^a^	Overall N = 21,418^a^
**Side**			
Left	7387 (50.5%)	3476 (51.2%)	10,863 (50.7%)
Right	7244 (49.5%)	3311 (48.8%)	10,555 (49.3%)
**Year of diagnosis**			
2014 – 2016	4157 (28.4%)	1475 (21.7%)	5632 (26.3%)
2017 – 2019^b^	4077 (27.9%)	1911 (28.2%)	5988 (28.0%)
2020 – 2022^b^	3760 (25.7%)	2036 (30.0%)	5796 (27.1%)
2023 – 2024	2637 (18.0%)	1365 (20.1%)	4002 (18.7%)
**Progesterone receptor status**			
Positive	12,222 (83.5%)	5719 (84.3%)	17,941 (83.8%)
**pT stage**			
1a	1686 (11.5%)	726 (10.7%)	2412 (11.3%)
1b	8100 (55.4%)	3823 (56.3%)	11,923 (55.7%)
1c	4829 (33.0%)	2227 (32.8%)	7056 (32.9%)
1, not further specified	16 (0.1%)	11 (0.2%)	27 (0.1%)
**Histological grade**			
Grade 1	10,197 (69.7%)	4632 (68.2%)	14,829 (69.2%)
Grade 2	4434 (30.3%)	2155 (31.8%)	6589 (30.8%)
**Morphology**			
No special type	11,886 (81.2%)	5241 (77.2%)	17,127 (80.0%)
Lobular carcinoma	1122 (7.7%)	569 (8.4%)	1691 (7.9%)
NST mixed with other tumour type	522 (3.6%)	221 (3.3%)	743 (3.5%)
Mucinous carcinoma	298 (2.0%)	340 (5.0%)	638 (3.0%)
Tubular carcinoma	456 (3.1%)	168 (2.5%)	624 (2.9%)
Other	347 (2.4%)	248 (3.7%)	595 (2.8%)
**Multifocality tumour**			
No	13,866 (94.8%)	6472 (95.4%)	20,338 (95.0%)
Yes	744 (5.1%)	306 (4.5%)	1050 (4.9%)
**DCIS besides the invasive tumour**	7159 (48.9%)	2964 (43.7%)	10,123 (47.3%)
**Surgeries needed for free margins**			
One lumpectomy	14,372 (98.2%)	6694 (98.6%)	21,066 (98.4%)
Two lumpectomies	259 (1.8%)	93 (1.4%)	352 (1.6%)
**Sentinel lymph node procedure performed**	14,603 (99.8%)	6765 (99.7%)	21,368 (99.8%)
**Endocrine therapy**	530 (3.6%)	212 (3.1%)	742 (3.5%)
**Radiation therapy**	13,894 (95.0%)	4488 (66.1%)	18,382 (85.8%)

a n (%).

b TOP-1 study enrolment period.

Clear surgical margins of the invasive component were achieved in a single procedure in 98.4% of patients. Re-excision was performed in 1.6%. A DCIS component, accompanying the invasive tumour, was present in 48.9% and 43.7% of the younger and older patients, respectively. Of the younger patients, 3.6% was treated with systemic ET, compared with 3.1% of older patients. RT was administered to 95.0% of younger patients versus 66.1% of older patients (Table 1). Baseline characteristics stratified per 10-year age groups are presented in Supplementary Table 1.

**Fig. 1:**
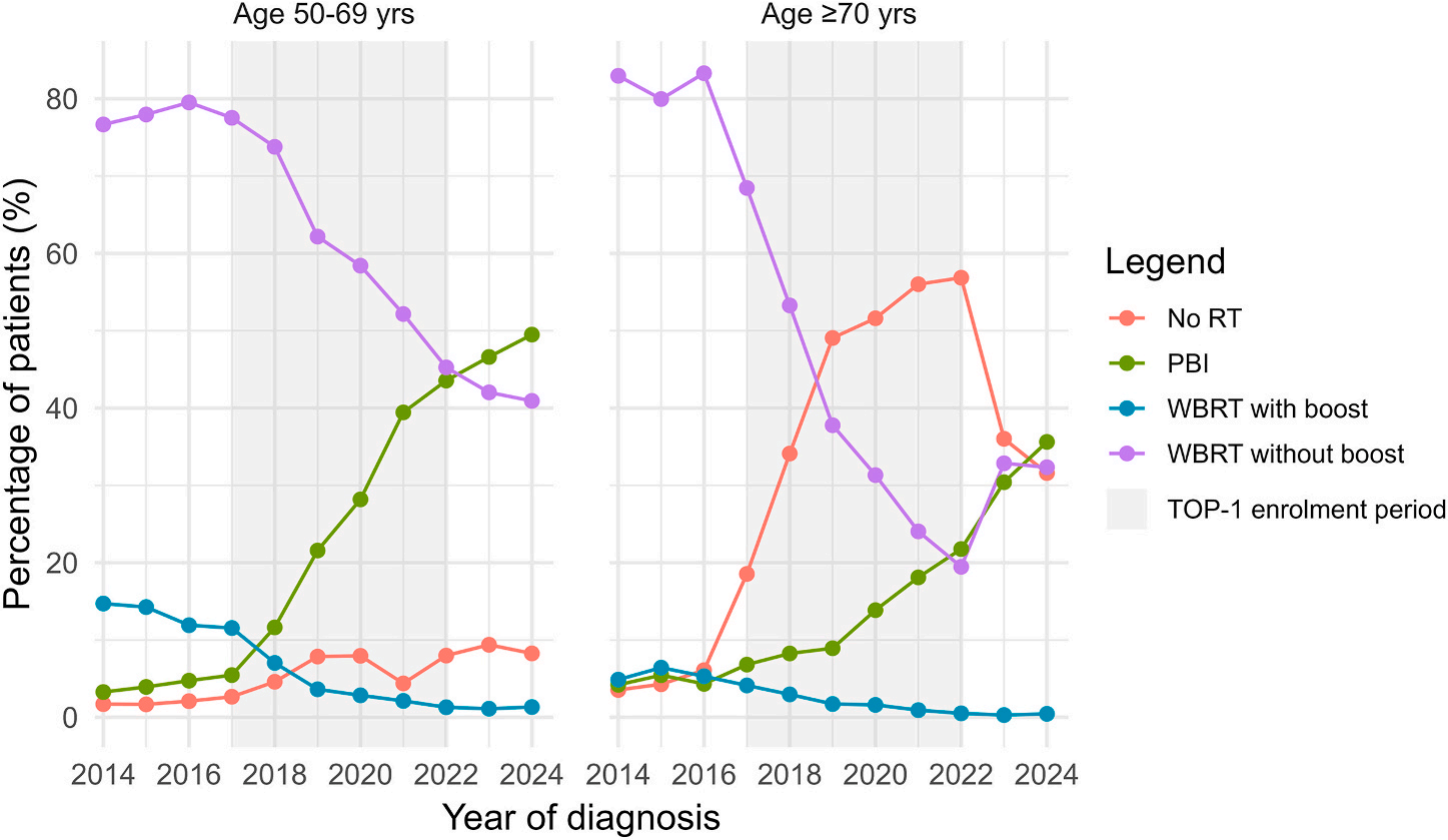
RT trends in younger and older patients.

During 2014-2016, RT was omitted in 2.5% of all patients, increasing to 13.9% in 2017-2019 and 23.6% in 2020-2022, thereafter decreasing to 17.3% in 2023-2024 (p < 0.001) (Fig. 1 and Supplementary Table 2). When looking at age groups stratified per 10-years, the decline in RT is the strongest in the oldest patients. In women aged 50-59 only 3.0% did not receive RT, rising to 6.4%, 31.6% and 56.3% in patients aged respectively 60-69, 70-79 and over 80 years old (p < 0.001).

Among patients who received RT, WBRT without boost was most common in all age groups, accounting for 65.8% in patients aged 50-69 years, 66.3% in 60-69 years, 70.5% in 70-79 years and 70.4% in those aged 80 years and older. PBI was the second most frequently used technique, with 21.8%, 26.3%, 23.4% and 23.7% in the respective 10-year age groups. The use of WBRT with boost was substantially lower, but relatively more often applied in younger patients (10.3% in those aged 50-59 years and 4.8% in 60-69 years) compared with older patients (3.7% in 70-79 and 2.2% in ≥ 80 years, p < 0.001).

Focussing on changes over time, the use of WBRT without boost declined from 81.2% in 2014-2016 to 46.5% in 2023-2024. In contrast, the use of PBI increased from 4.3% in 2014-2016 to 51.9% in 2023-2024. WBRT with boost was administered to a smaller proportion of patients compared with other RT types, and declined from 11.8% to 1.1% over time (p < 0.001). These proportional changes in RT type were comparable between both younger and older patients, as illustrated in Fig. 1.

**Fig. 2:**
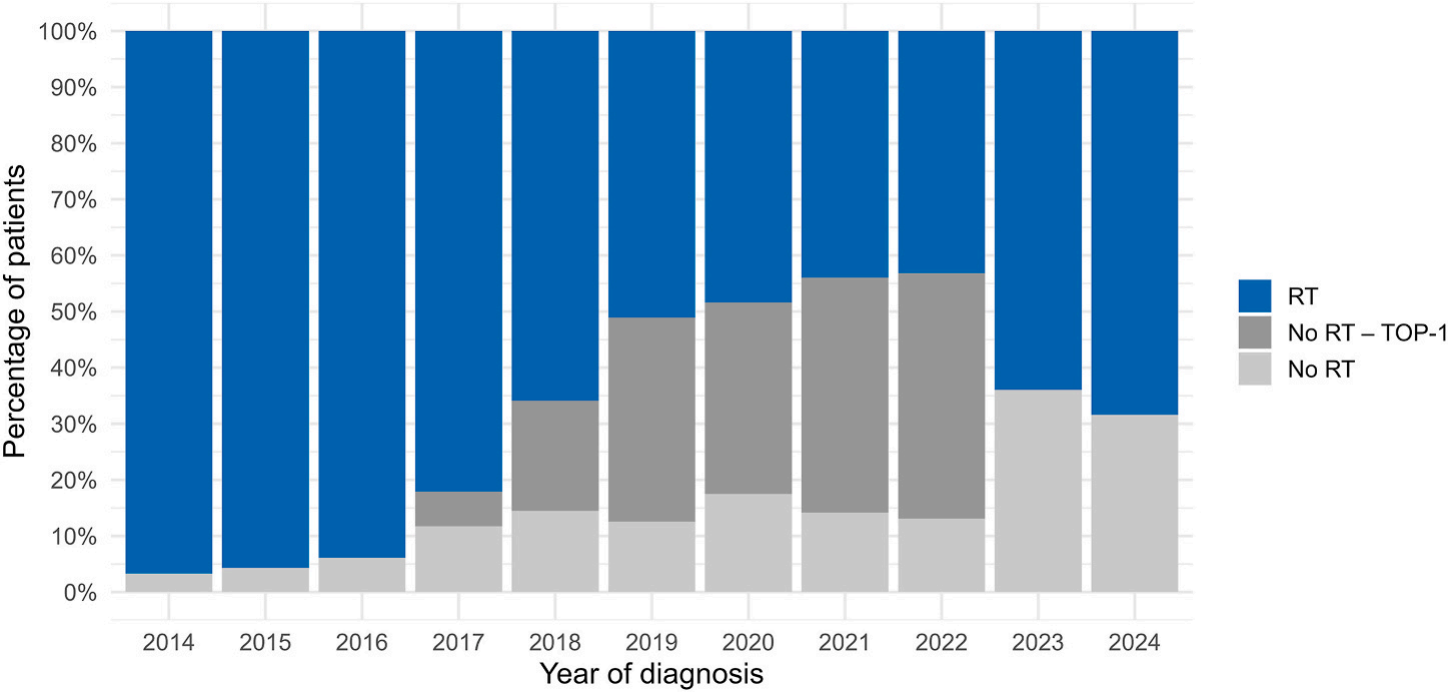
RT in older patients (≥70) over the years.

In 2014-2016, radiation therapy was omitted in 1.8% of younger patients. The omission rate increased over the years to 4.7% (2017-2019), 6.5% (2020-2022) and 8.7% (2023-2024), although the administration of RT remained above 90% in this younger population (p < 0.001) (Table 2 and Supplementary Fig. 1). Focussing on the older population (≥70 years), a clear temporal trend was observed in omission of RT (Fig. 2). In 2014-2016, RT was omitted in 4.6%, rising to 33.7% and 55.3% in 2017-2019 and 2020-2022, respectively, before declining again to 33.8% in 2023-2024 (Table 2). Between 2017 and 2022, patients from this older population were simultaneously included in the national TOP-1 study investigating RT omission after BCS. However, after the enrolment period ended and RT administration increased again to 66.2% in 2023-2024, omission rates remained higher than the 4.6% observed before study initiation.

**Table 2:** (Type of) radiation therapy over time stratified by younger and older patients.

Younger patients (50-69 years)										
Characteristic	By RT				By type of RT					
	Overall N = 14,631^a^	Yes N = 13,894^b^	No N = 737^b^	p-value^c^	Overall N = 13,871^a^	WBRT without boost N = 9,166^b^	Partial breast irradiation N = 3,391^b^	WBRT with boost N = 978^b^	Other N = 336^b^	p-value^d^
**Year of diagnosis**				<0.001						<0.001
2014 – 2016	4157	4084 (98.2%)	73 (1.8%)		4081	3246 (79.5%)	166 (4.1%)	565 (13.8%)	104 (2.5%)	
2017 – 2019^e^	4077	3887 (95.3%)	190 (4.7%)		3875	2911 (75.1%)	515 (13.3%)	307 (7.9%)	142 (3.7%)	
2020 – 2022^e^	3760	3516 (93.5%)	244 (6.5%)		3511	1915 (54.5%)	1444 (41.1%)	74 (2.1%)	78 (2.2%)	
2023 – 2024	2637	2407 (91.3%)	230 (8.7%)		2404	1094 (45.5%)	1266 (52.7%)	32 (1.3%)	12 (0.5%)	

**Older patients (≥ 70 years)**										
**Characteristic**	**Overall** **N = 6,787**^a^	**Yes** **N = 4,488^2^**	**No** **N = 2,299**^b^	**p-value**^c^	**Overall** **N = 4,482**^a^	**WBRT without boost** **N = 3,160**^b^	**Partial breast irradiation** **N = 1,048**^b^	**WBRT with boost** **N = 162**^b^	**Other** **N = 112**^b^	**p-value**^d^

**Year of diagnosis**				<0.001						<0.001
2014 – 2016	1475	1407 (95.4%)	68 (4.6%)		1406	1210 (86.1%)	69 (4.9%)	82 (5.8%)	45 (3.2%)	
2017 – 2019^e^	1911	1267 (66.3%)	644 (33.7%)		1262	1015 (80.4%)	153 (12.1%)	56 (4.4%)	38 (3.0%)	
2020 – 2022^e^	2036	911 (44.7%)	1125 (55.3%)		911	490 (53.8%)	376 (41.3%)	19 (2.1%)	26 (2.9%)	
2023 – 2024	1365	903 (66.2%)	462 (33.8%)		903	445 (49.3%)	450 (49.8%)	5 (0.6%)	3 (0.3%)	

a n.

b n (%).

c Pearson's Chi-squared test; Fisher's exact test.

d Pearson's Chi-squared test.

e TOP-1 study enrolment period.

Multivariable logistic regression analysis showed that older age and year of diagnosis were associated with RT omission (Table 3). The OR was 10.10 (95% CI 9.20-11.10) in patients aged ≥70 years compared to those aged 50-69 years. Odds of RT omission increased over time, with a peak in 2020-2022 (OR 13.30, 95% CI 11.10-16.10) and an OR of 8.39 (95% CI 6.94-10.20) in 2023-2024, though all periods remained significantly elevated compared to 2014-2016. Tumour characteristics negatively associated with omission of RT were pT1b and pT1c tumours (OR 0.68, 95% CI 0.59-0.78 and OR 0.53, 95% CI 0.46-0.62, respectively), grade 2 histology (OR 0.67, 95% CI 0.60-0.75), lobular carcinoma (OR 0.76, 95% CI 0.63-0.91), presence of DCIS (OR 0.83, 95% CI 0.76-0.91) and multifocality (OR 0.70, 95% CI 0.56-0.88).

**Table 3:** Multivariable logistic regression used to determine odds ratios and clinicopathological (baseline) factors associated with omission of radiation therapy.

Characteristic	OR^a^	95% CI^b^	p-value
**Age category**			
Age 50-69 yrs	Ref		
Age ≥70 yrs	10.10	9.20 – 11.10	**<0.001**
**Year of diagnosis**			
2014 – 2016	Ref		
2017 – 2019	6.38	5.30 – 7.73	**<0.001**
2020 – 2022	13.30	11.10 – 16.10	**<0.001**
2023 – 2024	8.39	6.94 – 10.2	**<0.001**
**pT stage**^c^			
1a	Ref		
1b	0.68	0.59 – 0.78	**<0.001**
1c	0.53	0.46 – 0.62	**<0.001**
**Histological grade**			
Grade 1	Ref		
Grade 2	0.67	0.60 – 0.75	**<0.001**
**Morphology**			
No special type	Ref		
Lobular carcinoma	0.76	0.63 – 0.91	**0.003**
Mucinous carcinoma	1.10	0.88 – 1.37	0.406
Tubular carcinoma	1.18	0.90 – 1.53	0.220
NST mixed with other tumour type	1.01	0.78 – 1.28	0.964
Other	1.18	0.92 – 1.51	0.178
**Progesterone receptor status**			
Negative	Ref		
Positive	1.00	0.89 – 1.13	0.955
**DCIS besides the invasive tumour**			
No	Ref		
Yes	0.83	0.76 – 0.91	**<0.001**
**Multifocality tumour**			
No	Ref		
Yes	0.70	0.56 – 0.88	**0.002**

a OR = Odds Ratio.

b CI = Confidence Interval.

c “1, not further specified” was excluded from the model due to very small numbers.

## 4 Discussion

In this cohort of women with early-stage BC, RT was administered more frequently in younger than in older patients. Similar trends in type of RT were seen over time in older and younger women. For all age categories, WBRT without boost was the most common RT modality, decreasing over the years, while the application of PBI increased. Both approaches were used at approximately equal frequencies in the last years. Over time, the administration of RT remained above 90% in younger women, although a modest yet statistically significant increase in RT omission was observed. The de-escalation trend was more pronounced in older patients, with a peak during the national TOP-1 study investigating omission of RT after BCS in this population. Age and year of diagnosis were the strongest predictors of RT omission. Larger tumour size, grade 2 tumours, lobular carcinoma, multifocality and presence of a DCIS component were associated with administration of RT. The increase in omission between 2017 and 2022 can partly be attributed to the TOP-1 study. However, the trend towards RT de-escalation continued, with omission reaching approximately one third of older patients in subsequent years, exceeding pre-study levels.

The type of RT following BCS depends, next to patient preferences, on tumour characteristics and risk factors such as grade, radicality, hormone receptor positivity and lymphovascular invasion [6,[22], [23], [24], [25], [26]]. Both PBI and WBRT decrease the risk of a LR [[26], [27], [28]]. PBI possibly causes less therapy burden as the targeted area is smaller compared with WBRT, and is associated with better cosmetic outcomes [26]. Initially, the treatment schedule for PBI was shorter, however, over time this difference has diminished as ultrahypofractionated WBRT schedules became standard practice in the Netherlands [6]. Similar LR rates and OS outcomes comparing PBI and WBRT were observed in selected patients with early-stage invasive BC [26,[28], [29], [30], [31]].

Regarding omission of RT, previous trials such as the PRIME II and CALBG 9343 have demonstrated that 5-year LR rates were low and OS was not negatively affected when WBRT was omitted after BCS in older patients [[8], [9], [10]]. These findings raise the question of whether a substantial proportion of older patients may be overtreated with RT. The risk of competing mortality from other causes than BC rises with increasing age [32,33]. When making a treatment decision, the individual risk of death from other causes and side effects must be taken into account, and this risk must be weighed against the risk of local disease recurrence.

Although LR rates without WBRT were low in these studies, it should be noted that the vast majority of patients were treated with ET [8,10,34]. In the Netherlands, guidelines are more conservative with no ET indicated for low-risk tumours [6,35,36]. The Dutch Breast Cancer Guidelines advices to omit ET for BC patients with a very good prognosis and low risk of recurrence, weighing a very small expected benefit without survival gain against treatment burden, adverse events and costs [6].

Internationally, most patients with low risk breast cancer do receive ET [37,38]. When RT is omitted in the presence of ET, ET compensates on LR risk through its systemic effect. ET diminishes the absolute benefit of postoperative RT, strengthening the rationale for de-escalation of locoregional treatment in older patients with low-risk BC. Conversely, in the absence of ET, the role of RT in local control may be more important, which should be considered when extrapolating our findings internationally, where ET is standard of care.

From February 2020 onwards, the Dutch Breast Cancer guidelines advice that WBRT omission may be considered in extremely low-risk BC patients (>70 years old, pT1a/b, grade 1 or 2, ER+, HER2-, N0, free margins), taking into account patient preferences, comorbidities and life expectancy [6]. The guideline explicitly acknowledges that this recommendation is mainly based on studies with concomitant ET and that supporting evidence is still limited [6,11]. The increase in RT omission in older patients in our study may be partly attributed to this addition. The previously mentioned TOP-1 study (results pending, CCMO NL58117.058.16) seeks to fill this knowledge gap, investigating omission after BCS in the absence of ET prospectively in patients aged ≥70 years. Furthermore, the EUROPA trial is researching ET versus RT after BCS in older luminal A BC patients and the Danish Natural trial compared PBI with no postoperative RT in patients aged ≥ 60 years old [39,40].

Personalization of treatment through well informed SDM, has become increasingly important over time and may also have contributed to the increase in RT de-escalation [8,10,34]. Both national and international studies have shown that SDM improved when decision-aid tools for BC were used [41,42]. The BRASA study, a Dutch study evaluating a RT decision-aid tool between 2017 and 2019, showed that patients chose less often for postoperative treatment after the use of the decision-aid tool. The authors observed a slightly better uptake of the decision-aid tool in younger patients [43]. This SDM tool may have contributed to the increased RT omission in younger patients.

A strength of this study is the nationwide scope of this article, enabling a clear and robust overview of the total population. Whereas the decrease in RT use has previously been described by Evers et al. who studied the period from 2009 up to 2019, our study adds to this work by focussing on a more selected patient population and capturing more recent years [15]. Furthermore, the data from the NCR are systematically collected from routine care, including all eligible patients across the country, thereby enhancing generalizability. The study also has limitations. No information about comorbidities, performance and frailty status were available in the dataset, nor DCIS grade or distance to narrowest margin, so considerations for certain treatment options are not clear. When DCIS grade 3 was present, this might have been an indication for WBRT with a boost [44]. In clinical practice, some patients may have received WBRT with boost because of this reason. Nevertheless, this study provides a clear overview of a selected clinically low-risk population. Linking these data to long-term outcomes such as recurrence and survival is an important next step. As the Netherlands is, as far as we know, the only country worldwide where these patients are not routinely treated with ET, results from the TOP-1 study are highly anticipated to shape international guidelines and strengthen the evidence base for SDM.

## 5 Conclusion

In patients aged 50 years and older with early-stage hormone receptor-positive BC, substantial changes in RT were observed over the past decade. Younger patients were more often treated with RT compared with older patients. The use of PBI following BCS increased, while WBRT rates declined. Omission of postoperative RT increased over time in both age groups, but was most evident in women aged 70 years and older, although the safety of omission of RT in the absence of ET is yet to be established. The underlying reasons for this de-escalation trend may reflect a combination of evolving guidelines, increased SDM, and the influence of a national ongoing trial, possibly with premature implementation into clinical practice.

## Disclosure

The authors declare no conflict of interest and have no financial support to disclose.

## Ethics

This is an observational study using data from the Netherlands Cancer Registry. The privacy board of the Netherlands Comprehensive Cancer Organisation (IKNL) gave permission to use these data (request 25-00948). A non-WMO declaration has been issued by the Leiden University Medical Center's non-WMO committee.

## Funding

This research received no funding.

## CRediT authorship contribution statement

**Lotte Bouwman:** Conceptualization, Formal analysis, Methodology, Visualization, Writing – original draft, Writing – review & editing. **Erik J. Blok:** Formal analysis, Methodology, Supervision, Visualization, Writing – review & editing. **Astrid N. Scholten:** Methodology, Supervision, Writing – review & editing. **Sabine Siesling:** Methodology, Supervision, Writing – review & editing. **Johanneke E.A. Portielje:** Conceptualization, Methodology, Supervision, Visualization, Writing – original draft, Writing – review & editing. **Gerrit-Jan Liefers:** Conceptualization, Methodology, Supervision, Visualization, Writing – original draft, Writing – review & editing.

## Declaration of competing interest

The authors declare that they have no known competing financial interests or personal relationships that could have appeared to influence the work reported in this paper.
