# Supplementary material for: Radiation therapy trends following breast-conserving surgery in early-stage estrogen receptor-positive breast cancer: particularly in older patients more frequently omitted
Source: Breast. 2026 Aug 5;89:104893. doi: 10.1016/j.breast.2026.104893 (PMC13487387; doi:10.1016/j.breast.2026.104893)
Supplement: Supplementary file 1 [file mmc1.docx]

## Supplementary

Table 1. Baseline characteristics stratified per ten-year age groups

| **Characteristic** | **Age 50-59 yrs  N = 5,871^1^** | **Age 60-69 yrs  N = 8,760^1^** | **Age 70-79 yrs  N = 6,169^1^** | **Age ≥80 yrs  N = 618^1^** | **Overall  N = 21,418^1^** |
| --- | --- | --- | --- | --- | --- |
| **Side** |  |  |  |  |  |
| Left | 2,943 (50.1%) | 4,444 (50.7%) | 3,173 (51.4%) | 303 (49.0%) | 10,863 (50.7%) |
| Right | 2,928 (49.9%) | 4,316 (49.3%) | 2,996 (48.6%) | 315 (51.0%) | 10,555 (49.3%) |
| **Years around TOP-1** |  |  |  |  |  |
| 2014 – 2016 | 1,696 (28.9%) | 2,461 (28.1%) | 1,353 (21.9%) | 122 (19.7%) | 5,632 (26.3%) |
| 2017 – 2019* | 1,664 (28.3%) | 2,413 (27.5%) | 1,739 (28.2%) | 172 (27.8%) | 5,988 (28.0%) |
| 2020 – 2022* | 1,529 (26.0%) | 2,231 (25.5%) | 1,841 (29.8%) | 195 (31.6%) | 5,796 (27.1%) |
| 2023 – 2024 | 982 (16.7%) | 1,655 (18.9%) | 1,236 (20.0%) | 129 (20.9%) | 4,002 (18.7%) |
| **Progesterone receptor status** |  |  |  |  |  |
| Positive | 5,066 (86.3%) | 7,156 (81.7%) | 5,187 (84.1%) | 532 (86.1%) | 17,941 (83.8%) |
| Negative | 804 (13.7%) | 1,602 (18.3%) | 980 (15.9%) | 86 (13.9%) | 3,472 (16.2%) |
| **pT stage** |  |  |  |  |  |
| 1a | 640 (10.9%) | 1,046 (11.9%) | 686 (11.1%) | 40 (6.5%) | 2,412 (11.3%) |
| 1b | 3,153 (53.7%) | 4,947 (56.5%) | 3,572 (57.9%) | 251 (40.6%) | 11,923 (55.7%) |
| 1c | 2,075 (35.3%) | 2,754 (31.4%) | 1,903 (30.8%) | 324 (52.4%) | 7,056 (32.9%) |
| 1, not further specified | 3 (0.1%) | 13 (0.1%) | 8 (0.1%) | 3 (0.5%) | 27 (0.1%) |
| **Histological grade** |  |  |  |  |  |
| Grade 1 | 4,177 (71.1%) | 6,020 (68.7%) | 4,162 (67.5%) | 470 (76.1%) | 14,829 (69.2%) |
| Grade 2 | 1,694 (28.9%) | 2,740 (31.3%) | 2,007 (32.5%) | 148 (23.9%) | 6,589 (30.8%) |
| **Morphology** |  |  |  |  |  |
| No special type | 4,836 (82.4%) | 7,050 (80.5%) | 4,765 (77.2%) | 476 (77.0%) | 17,127 (80.0%) |
| Lobular carcinoma | 430 (7.3%) | 692 (7.9%) | 538 (8.7%) | 31 (5.0%) | 1,691 (7.9%) |
| NST mixed with other tumour type | 220 (3.7%) | 302 (3.4%) | 203 (3.3%) | 18 (2.9%) | 743 (3.5%) |
| Mucinous carcinoma | 73 (1.2%) | 225 (2.6%) | 289 (4.7%) | 51 (8.3%) | 638 (3.0%) |
| Tubular carcinoma | 203 (3.5%) | 253 (2.9%) | 160 (2.6%) | 8 (1.3%) | 624 (2.9%) |
| Other | 109 (1.9%) | 238 (2.7%) | 214 (3.5%) | 34 (5.5%) | 595 (2.8%) |
| **Multifocality tumour** |  |  |  |  |  |
| No | 5,517 (94.0%) | 8,349 (95.4%) | 5,875 (95.3%) | 597 (96.6%) | 20,338 (95.0%) |
| Yes | 348 (5.9%) | 396 (4.5%) | 285 (4.6%) | 21 (3.4%) | 1,050 (4.9%) |
| **DCIS besides the invasive tumour** | 3,036 (51.7%) | 4,123 (47.1%) | 2,716 (44.0%) | 248 (40.1%) | 10,123 (47.3%) |
| **Surgeries needed for free margins** |  |  |  |  |  |
| One lumpectomy | 5,750 (97.9%) | 8,622 (98.4%) | 6,082 (98.6%) | 612 (99.0%) | 21,066 (98.4%) |
| Two lumpectomies | 121 (2.1%) | 138 (1.6%) | 87 (1.4%) | 6 (1.0%) | 352 (1.6%) |
| **Sentinel lymph node procedure performed** | 5,856 (99.7%) | 8,747 (99.9%) | 6,151 (99.7%) | 614 (99.4%) | 21,368 (99.8%) |
| **Systemic endocrine therapy** | 252 (4.3%) | 278 (3.2%) | 180 (2.9%) | 32 (5.2%) | 742 (3.5%) |
| **Postoperative radiation therapy** | 5,695 (97.0%) | 8,199 (93.6%) | 4,218 (68.4%) | 270 (43.7%) | 18,382 (85.8%) |
| ^1^n (%), * TOP-1 study enrolment period | | | | | |

Figure 1. Radiation therapy in younger patients over time

Table 2. (Type of) radiation therapy over time and per age group

|  | **By RT*** | | | | **By RT type** | | | | | |  |
| --- | --- | --- | --- | --- | --- | --- | --- | --- | --- | --- | --- |
| **Characteristic** | **Overall  N = 21,418^1^** | **Yes  N = 18,382^2^** | **No  N = 3,036^2^** | **p-value^3^** | **Overall  N = 18,353^1^** | **WBRT without boost  N = 12,326^2^** | **Partial breast irradiation  N = 4,439^2^** | **WBRT with boost  N = 1,140^2^** | **Other  N = 448^2^** | **p-value^3^** |  |
| **Year of diagnosis** |  |  |  | <0.001 |  |  |  |  |  | <0.001 |  |
| 2014 – 2016 | 5,632 | 5,491 (97.5%) | 141 (2.5%) |  | 5,487 | 4,456 (81.2%) | 235 (4.3%) | 647 (11.8%) | 149 (2.7%) |  |  |
| 2017 – 2019** | 5,988 | 5,154 (86.1%) | 834 (13.9%) |  | 5,137 | 3,926 (76.4%) | 668 (13.0%) | 363 (7.1%) | 180 (3.5%) |  |  |
| 2020 – 2022** | 5,796 | 4,427 (76.4%) | 1,369 (23.6%) |  | 4,422 | 2,405 (54.4%) | 1,820 (41.2%) | 93 (2.1%) | 104 (2.4%) |  |  |
| 2023 – 2024 | 4,002 | 3,310 (82.7%) | 692 (17.3%) |  | 3,307 | 1,539 (46.5%) | 1,716 (51.9%) | 37 (1.1%) | 15 (0.5%) |  |  |
| **Age per 10 years** |  |  |  | <0.001 |  |  |  |  |  | <0.001 |  |
| Age 50-59 yrs | 5,871 | 5,695 (97.0%) | 176 (3.0%) |  | 5,686 | 3,740 (65.8%) | 1,239 (21.8%) | 583 (10.3%) | 124 (2.2%) |  |  |
| Age 60-69 yrs | 8,760 | 8,199 (93.6%) | 561 (6.4%) |  | 8,185 | 5,426 (66.3%) | 2,152 (26.3%) | 395 (4.8%) | 212 (2.6%) |  |  |
| Age 70-79 yrs | 6,169 | 4,218 (68.4%) | 1,951 (31.6%) |  | 4,212 | 2,970 (70.5%) | 984 (23.4%) | 156 (3.7%) | 102 (2.4%) |  |  |
| Age ≥80 yrs | 618 | 270 (43.7%) | 348 (56.3%) |  | 270 | 190 (70.4%) | 64 (23.7%) | 6 (2.2%) | 10 (3.7%) |  |  |
| ^1^n, ^2^n (%), ^3^Pearson's Chi-squared test, * RT = postoperative radiation therapy, ** TOP-1 study enrolment period | | | | | | | | | | | |
